# On the Necessity of Accoucheurs

**Published:** 1815-12

**Authors:** John Wayte


*For the London Medical and Physical Journal.*

### *On the Necessity of Accoucheurs;* by JOHN WAYTE, Esq.

IN Dr. Kinglake's paper on Morbid Infection, there is every thing to be admired, as bringing forth a subject to public view that will strike every practitioner as meriting a most attentive investigation, and which, I think, will meet it. But his paper in the last Number of your Journal, on Obstetric Practice, in my opinion, is altogether an opposite production. The practice of midwifery is a wide field of science: it embraces the whole period of pregnancy and after-treatment, as well as parturition; and, whether the doctor, in his term Practice of Midwifery, means to consign the whole over to female hands, I know not; but, if he solely urges the moment of labour, I will endeavour to prove that there are "legitimate objects" sufficient to justify the practice of midwifery remaining in the hands of male practitioners.

Without the least irreverance, or without detracting from the powers and perfections of nature, I do suppose and know that she is inadequate to accomplish every requisite at all times respecting the birth of human species; and that the deviations, mechanical as well as physiological, which not unfrequently shew themselves, warrant a constant attendance, and often a watchful eye over labours. Have we not occasionally a placenta presentation, where the os uteri must be artificially dilated, membranes ruptured, or placenta perforated, and the child turned and delivered quickly in order to insure the mother's life. Must we not at times assist in dilating a rigid os uteri? Is there ever a face-case at full time but what requires its axis to be shortened as much as possible, by keeping the chin low down? And are profuse uterine hæmorrhages after delivery so rare as not to speak for themselves, and pronounce their danger? Again, I would ask, how came the practice of midwifery to pass from the hands of women into the hands of men? Undoubtedly not from delicacy, and I think not from fashion, but from that strong reason—necessity. Midwives ill versed in the anatomy and physiology of all those parts concerned in parturition, and likewise of the human frame throughout, must have committed blunders and made difficulties where none existed; and, being incapable of removing them, or of overcoming those naturally present, were obliged to require aid from professional men, whose attendance assuredly would be solicited at future labours, in case of new impediments.

What

What was at first only occasional, in process of time became habitual; and here I will venture to say, that there is not one woman in a hundred throughout the kingdom, who, if able to afford it, will not prefer a male practitioner on those occasions; and why? because they consider themselves more safe. Are any persons so capable, and hence so proper, to be employed on obstetrical occasions, where, though nature is so generally all-sufficient, yet where it is universally admitted dangers in various degrees occasionally attend? I say, are any so capable as those whose whole study is the human structure, its physiology, reciprocal actions, dependencies, and morbid alterations? Who knows when and in what manner to counteract or assist Nature in her operations; and when to deem her all-sufficient. In easy natural labours, which Providence has made most numerous, the accoucheur has only to lend a few precautionary aids; but, as every day has brought, and will continue to bring, deviations, which require more patience, more manual assistance, and sometimes instrumental, so it was and is esteemed most prudent to have male attendants. Does the doctor think women preferable, through their ignorance and inadequacy, to difficulties? or men improper, because well versed in the whole department? Ignorance is surely much more prone to blunder, than science to be over-officious.

No one can too strongly reprobate undue instrumental aid: the man who resorts to it without sound reasons, and positive assurances of Nature's insufficiency, ought to be cashiered for ever: and I should hope, for the honour of our profession, that there are very few to be found so dead to their own good name, and the dictates of humanity, as wantonly to increase the sufferings of a fellow-creature. It may easily be said, that " bad cases in midwifery are announced by features not to be mistaken; that their urgency is not so great, but that there is time enough to call in special assistance;" and that this may be rendered by a neighbouring surgeon These positions, which have not experience for their guide, I must beg leave to dissent from. Midwives, if not considered, generally consider themselves expert, yet often make most egregious blunders: they have mistaken, and might again mistake, a hand for a foot, and, insensible of error, perseveringly draw down. The consequence would be that their aid would be injurious, at least ineffectual, and medical assistance be required; this from the distance of two to ten miles, all which time the patient suffers bodily as well as mentally;—the uterus in vain continues to perform its part towards expulsion, and the accoucheur, whose first attendance might have accomplished delivery with comparative

 ease, ease, has now to contend with increased difficulties. Other cases could be alleged quite as pertinent, but which I deem intrusive. I must, therefore, enter my protest against man-midwifery being confined to "absolute occasions for operative aid," and beg that it might remain in those hands in which necessity, prudence, and other causes, have concurred in placing it, and under which it has arrived at its present perfection; where neither "the interests of hnmanity, dignity of science, or cause of truth," are in the least degraded, or the patients comfort at all molested; neither is it gratuitous, meretricious, hurtful, busy, intermeddling, nor mischievous."

*Calne; Oct.* 26, 1815.

